# Sporotrichosis in Iowa

**Published:** 1913-11

**Authors:** 


SPOROTRICHOSIS IN IOWA. The first case recorded was reported by Albert and Grover, the second editorially, in the *Iowa Med. Jour.,* March and July, 1913, respectively.

